# **World Kidney Day 2011: Protect Your Kidneys, Save Your Heart**

**Published:** 2011-02-01

**Authors:** Sara Martin, W. G. Couser, M. C. Riella

**Affiliations:** 1 For the Joint International Society of Nephrology (WGC) and International Federation of Kidney Foundations (MCR) World Kidney Day 2011 Steering Committee*; 2 *Willliam G Couser (ISN), Miguel C Riella (IFKF), Co-Chairmen. Georgi Abraham, Paul Beerkens, John Feehally, Guillermo Garcia Garcia, Jan Lantik, Dan Larsen, Philip Li, Mark Murphy and Bernardo Rodriguez-Iturbe.*

**INTRODUCTION TO WORLD KIDNEY DAY 2011**

March 10, 2011 will mark the celebration of the 6^th^ World Kidney Day (WKD), an annual event jointly sponsored by the International Society of Nephrology and the International Federation of Kidney Foundations. Since its inception in 2006, WKD has grown dramatically to become the most widely celebrated event associated with kidney disease in the world and the most successful effort to raise awareness among both the general public and government health officials about the dangers of kidney disease, especially chronic kidney disease (CKD). 

In 2011, WKD will call attention to the large, and often unappreciated, role played by kidney dysfunction in increasing premature cardiovascular disease, the most common cause of morbidity and mortality worldwide [1].

Can a focus on early detection and prevention of kidney disease really improve long-term cardiovascular health? In this editorial, we hope to convey the message that increased attention to the kidneys can indeed improve long-term health outcomes by reducing both kidney and cardiovascular disease and should therefore be a central component of any global health strategy intended to reduce the enormous and growing burden of chronic non-communicable diseases (NCDs).

**CARDIOVASCULAR DISEASE (CVD) AND THE KIDNEY**

CVD is the most common of the chronic NCDs that impact global mortality. About 30% of all deaths worldwide and 10% of all healthy life lost to disease are accounted for by CVD alone [1]. Although there has been some decline in mortality from CVD in developed countries, no such decline has been reported in developing countries, ethic and socially disadvantaged minority populations or in people with accompanying CKD [2,3].

The presence of CKD significantly increases the risk of a CV event in both diabetes and hypertension [4,5]. However, less well appreciated is that CKD alone is a strong risk factor for CVD, independent of diabetes, hypertension or any other conventional CVD risk factor [6,7]. This is especially true when an increase in proteinuria, a major target of any CKD screening program, is present [6-9]. 

The 20–30-fold increase in CVD in patients with end-stage renal disease (ESRD) has long been recognized, but the increased risk for CVD associated with lesser degrees of renal functional impairment was definitively demonstrated only in 2004. Go, *et al*, reported an independent and graded association between glomerular filtration rate (GFR) and risk of death, cardiovascular (CV) events and hospitalizations in a community-based study of over 1.4 million individuals [6]. 

Is this dramatic increase in CVD risk associated with CKD really due to CKD or does it just reflect the coexistent diabetes or hypertension that are present in a majority of these patients? The independent effect of CKD alone has now been well documented in many studies [7]. The risk of cardiac death is increased 46% in people with a GFR between 60 and 90 mLI/min and 131% in those with a GFR between 30 and 60 mLI/min (stage III CKD) independent of traditional CV risk factors including diabetes and hypertension [10]. The increased risk for CV events and mortality in people over 55 with CKD alone is equivalent, or even higher, to that seen in patients with diabetes or previous myocardial infarcts [11].Both general [6,12] and high-risk populations [13,14] exhibit an increased risk of CVD with CKD. This increased risk for CVD is not confined to the elderly—in volunteers with an average age of 45, the risk for myocardial infarct, stroke and all cause mortality was doubled in those with CKD [14]. 

**PROTEINURIA AND CV RISK**

In considering the value of recommending screening for CKD along with conventional CVD risk factors in selected individuals data showing that the risk of CVD is better correlated with proteinuria (albuminuria), than with GFR alone is particularly relevant because proteinuria is virtually always a marker of kidney disease and is not a conventional CVD risk factor [6,8,9,15].

With regard to proteinuria as a predictor of later CVD, The PREVEND study showed a direct linear relationship between albuminuria and risk of CV death in the general population even at levels of albumin excretion generally considered within the “normal” range (15–29 mg/day) and was increased more than six-fold when albumin excretion exceeded 300 mg/day [8]. 

Recent data from the US NHANES database as well as from Japan also document an independent effect of albuminuria on risk of both CVD and all cause mortality at all levels of GFR [15,16]. In patients with congestive heart failure but without diabetes or hypertension, increased urinary albumin predicts both CV and all cause mortality independent of reduced GFR [17]. Similar results are obtained studying patients with coronary disease or previous myocardial infarcts in whom proteinuria conferred a greater risk of mortality than reduced GFR, although both adversely impacted outcomes [18].

Of interest, not only the likelihood but also the time to development of a CV event is accelerated significantly by the presence of proteinuria at all levels of GFR [19]. In non-diabetic subjects with normal serum creatinine levels undergoing percutaneous coronary interventions, about 78% have demonstrable CKD when screened more stringently for eGFR, urine protein [20]. Not only is the presence of CKD a likely factor in accelerating development of coronary disease in these patients but it has also been associated with an increase in other risks including hemorrhagic complications, contrast nephropathy, re-stenosis, and death [10]. Thus, multiple studies now confirm that proteinuria is a graded risk factor for CVD independent of GFR, hypertension and diabetes and that this risk extends down into ranges of albumin excretion generally considered “normal” [21,22]. Moreover, this increased CV risk has been well demonstrated in several studies where only dipsticks were used to screen for increased protein excretion [6,18,23]

Although there has been concern that CKD diagnosed by reduced GFR alone identifies predominately older adults at increased risk because of age alone [24], the connection between proteinuria as an independent risk factor for CV mortality has been confirmed by meta-analysis of 22 separate, general population, cohort studies and in both older (>65 years) and younger people of several nationalities and racial groups [23].

**CAN TREATMENT OF CKD REDUCE CVD?**

Finally, and most importantly from a clinical perspective, there is provocative data to suggest that renal-targeted interventions designed to reduce proteinuria and slow progression of CKD can reduce CVD risk as well. Angiotensin-converting enzyme inhibitors (ACEIs) and/or angiotensin receptor blockers (ARBs) are of documented benefit in slowing progression of established diabetic and non-diabetic CKD [25-29]. Of interest related to slowing progression, the incidence of CVD in CKD is significantly higher with more rapid loss of GFR independent of other risk factors, suggesting that interventions that slow progression may also reduce CVD [19]. PREVEND IT reported a 40% (although not significant) reduction in CV events over four years in patients screened from a general population with no risk factors except increased albumin in the urine who were treated with renal-targeted ACEI therapy [30]. In this pilot study, this effect was seen primarily in people with albumin excretion rates of >50 mg/day, and the intervention was shown to be cost-effective in that population [31]. In the RENNAL study CV endpoints were significantly reduced in direct proportion to the reduction of albuminuria with ACEI therapy in type 2 diabetics, and albuminuria proved to be the only predictor of CV outcome [32]. The potential benefit of renal-targeted therapies has recently been highlighted by observations that higher doses of renin-angiotensin system (RAS) blockers than required for BP control alone can further reduce proteinuria independent of effects on BP or GFR, and that addition of salt restriction or diuretics, both very inexpensive interventions, can further enhance the proteinuria-reducing effect of RAS blockade [33,34]. Data are not yet available to establish that screening for CKD and subsequent interventions will reduce CV mortality and be cost-effective in younger people (<55 years) [35]. However, it is now known that albuminuria is a better predictor of renal and cardiovascular events than blood pressure alone, that reducing proteinuria is more renal and cardio protective than lowering blood pressure alone and that identification of CKD can improve CV outcomes. 

## CONCLUSION

As celebrations of the 6^th^ WKD approach on March 10, 2011, it is worth noting that prior to the past decade, kidney disease was seen by most government and public health authorities as largely confined to patients with ESRD, thankfully a rare condition because the enormous cost of renal replacement therapy disproportionately consumes scarce health care resources and is well beyond the means of countries inhabited by over 80% of the worlds population [36,37]. Much has changed. We now appreciate that kidney disease is not rare—some 10% of the population has evidence of renal dysfunction. And, we know these individuals are not of concern just because a few will progress to ESRD, but more because they carry a greatly enhanced risk of premature death from CVD, the single largest and most expensive health care threat we confront at a global level [1]. Just as progress is being made in treating most of the traditional CV risk factors, CKD has emerged as yet another one that causes substantial vascular toxicity independently. Fortunately, there is good news as well. Biomarkers of CKD (proteinuria, eGFR) are easy and relatively inexpensive to detect, and one of these, proteinuria, emerges early in the evolution of generalized vascular disease. Thus, kidney-targeted detection and prevention programs seem to offer a valuable opportunity to institute early preventive measures that go beyond traditional cardio-protective approaches. There is now compelling evidence that including selective screening for CKD in global health programs designed primarily to reduce CVD will significantly improve the outcomes of not only renal disease, but especially the NCDs like diabetes and CVD that dominate future health care strategies. Roadmaps for accomplishing this have already been presented for both developed [38,39] and emerging [1,40] countries. However, effective implementation of such strategies will only come when both the general public and the renal community work together to convince health authorities that it is in the public interest to do this. It is our sincere hope that worldwide celebration of WKD 2011 will provide an opportunity to reinforce the message that kidney disease is indeed common, harmful and treatable and that protecting your kidneys is an important health strategy that may save your heart.
